# Mast cell gastritis: Children complaining of chronic abdominal pain with histologically normal gastric mucosal biopsies except for increase in mast cells, proposing a new entity

**DOI:** 10.1186/1746-1596-4-34

**Published:** 2009-10-03

**Authors:** Fatemeh E Mahjoub, Fatemeh Farahmand, Zahra Pourpak, Hoda Asefi, Zahra Amini

**Affiliations:** 1 Pathology Department, Children Medical Center, Tehran University of Medical Sciences, Tehran, Iran; 2 Pediatric Gastroenterology Department, Children Medical Center, Tehran University of Medical Sciences, Tehran, Iran; 3 Immunology, Asthma and Allergy Research Center, Children Medical Center, Tehran University of Medical Sciences, Tehran, Iran; 4 Tehran University of Medical Sciences, Tehran, Iran

## Abstract

**Background:**

Mast cells reside within the connective tissue of a variety of tissues and all vascularized organs. Since 1996, few studies have been performed on mast cell density in gastrointestinal biopsies, mainly in adult age group. We recently studied mast cell density in pediatric age group on rather larger number of cases in a referral children hospital. Mast cell density was 12.6 ± 0.87 in 0.25 mm^2 ^(range: 0-81) in our study.

Since we frequently encounter cases with rather normal gastric biopsies with no H.pylori, which mainly complain of chronic abdominal pain, we gathered those cases with mast cell density more than 30/0.25 mm^2^. from 895 gastric biopsies and wanted to study their clinical and endoscopic findings and propose a new entity.

**Methods:**

Between April 2005 and May 2008, 895 children (< 14 years old), with gastrointestinal complaints who underwent endoscopy were selected and antral biopsies were obtained for histological examination. Among these children, those who had normal or erythematous (but not nodular or ulcerative) gastric mucosa on endoscopic view, plus pathologic report of normal mucosa or mild gastritis in addition to mast cell count more than 30/25 mm^2^, were chosen and a questionnaire was filled for each patient including clinical, endoscopic and pathologic findings.

The statistical analysis was performed using SPSS, version 13 (SPSS Inc., Chicago, IL, USA).

**Results:**

Over a 3 year period of study, of 895 selected children, 86 patients fulfilled the entrance criteria. The major complaint of patients was recurrent abdominal pain. The mean mast cell density was 45.59 ± 13.81 in 0.25 mm^2 ^(range: 30-93). Among our cases, about 67.4% (n = 58) had 30 to 49, 23.3% (n = 20) had 50 to 69, 8.1% (n = 7) had 70 to 89 and 1.2% (n = 1) had 93 mast cells/0.25 mm^2 ^in their specimens

**Discussion:**

In 29% of our cases, neither endoscopic nor pathologic change was detected and only increase in mast cell number was reported and in others endoscopic and histopathological findings were negligible except increase in mast cells. In updated Sydney system (classification and grading of gastritis), no term is introduced which is in concordance with this group but we think that increased density of mast cells in these cases should not be overlooked and it may contribute to clinical manifestations in some way. We hope that further studies will direct us to institute therapeutic measurements in this regard.

## Introduction

Mast cells are round, generally mononuclear cells, which exhibit phenotypic variation in morphology. Cell diameter ranges up to 25 μm. Nucleus is unilobed and may be round or oval in shape and is typically eccentrically positioned. Electron microscopy shows numerous cytoplasmic projections that may interdigitate with other cells [1].

**Figure 1:**
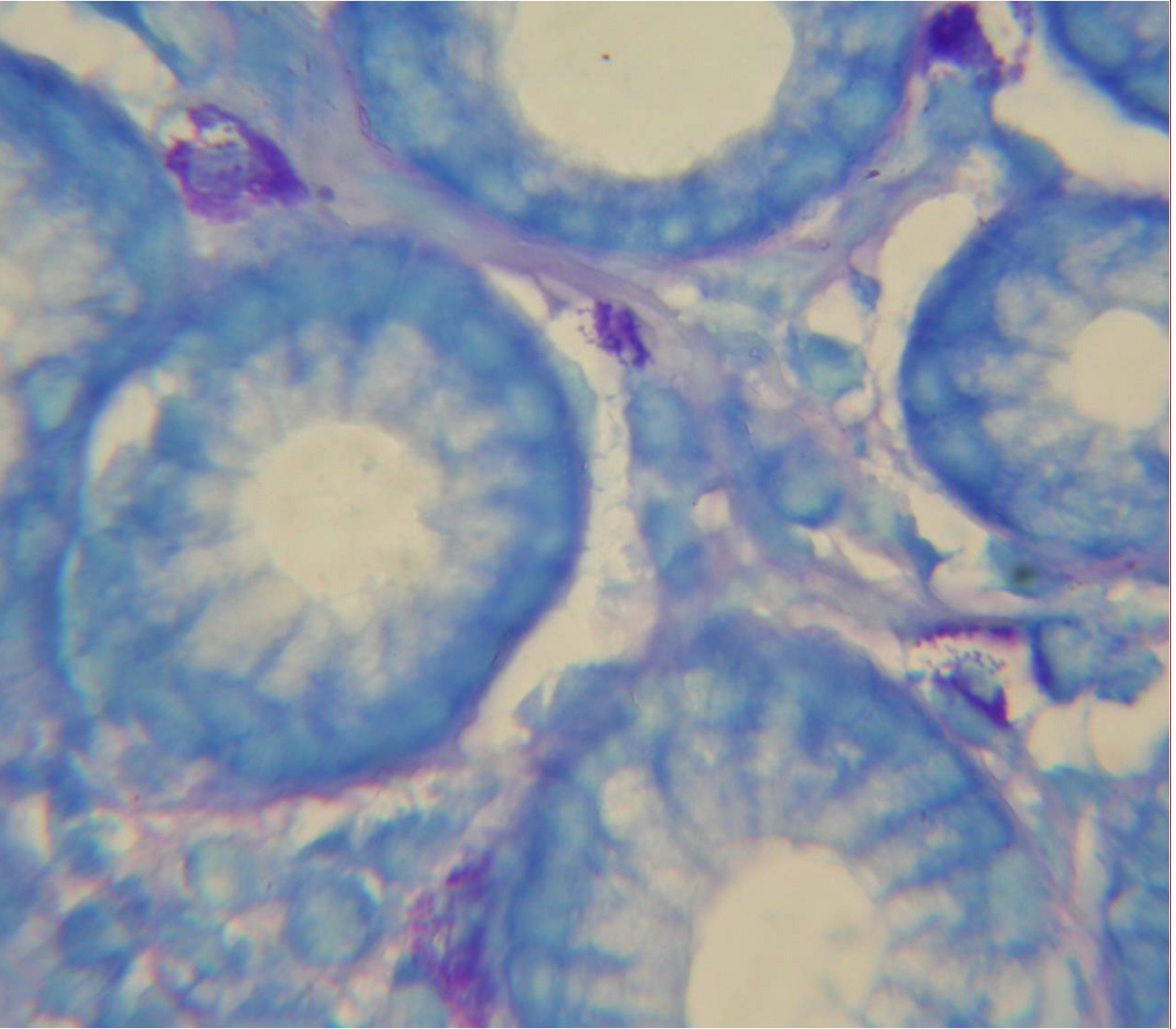
**Mast cells are stained by Giemsa staining and are seen best by oil (×1000), in this case five mast cells are seen in this high power field**.

Mast cell can be recognized by its numerous metachromatic cytoplasmic granules, which range in size from 0.3 to 0.8 μm and which may occupy the majority of the cell volume [1]. These granules stain red-purple with the basic dye toluidine blue or Giemsa (figure 1). This property of staining red upon application of a blue dye is referred to as metachromatic staining and is due to the presence of highly sulfated, anionic proteoglycans complexed with the various secretory granule proteases, a property that is shared by basophils. Poorly granulated mast cells or mast cells that have recently degranulated extensively, if stained with Giemsa or toluidine blue, can be mistaken for eosinophils or histiocytes, a detail that can complicate the diagnosis of mast cell related disorders [1-7].

Mast cells reside within the connective tissue of a variety of tissues and all vascularized organs. Their numbers and densities are highest at interfaces between the internal and external environments where they can respond to foreign organisms and antigens [1]. Sites include the dermis, gut mucosa and submucosa, conjunctiva, pulmonary alveoli and airways, and the atrial appendage of the heart [1-7].

Since 1996, few studies have been performed on mast cell density in gastrointestinal biopsies, mainly in adult age group. Sulik et al. studied mast cell involvement in children with or without infection by Helicobacter pylori (H.pylori). Results of this study showed that mast cell through its numerous mediators may play a key role in chronic gastritis especially in H. pylori positive cases [8]. Mysorekar et al have reported that mast cell counts were significantly higher in the antral mucosa in gastritis [9]. Nakajima et al demonstrated the presence of mast cells by immunohistochemical staining with anti human tryptase antibody in gastric biopsies and concluded that mast cells may be important effector cells in the pathogenesis of gastritis [10]. Fazilet Kayaselcuk et al investigated the relationship between mast cell density, H. pylori density and histopathological severity of gastritis in the corpus and antral mucosa and concluded that the difference between the mast cell density in the antrum and that in the corpus was significant for all three variables [11]. N. Moorchung et el attempted to study the relationship between endoscopy and the mast cell and eosinophil infiltrate and also studied the role of gene polymorphisms, Helicobacter pylori density and the CagA antibody status in influencing the mast cell and eosinophil infiltrate, but in contrast to other studies, mast cells did not have a significant role in their study [12].

As mentioned above, studies on mast cell density have been performed mainly on adults and on rather small groups as yet. We recently studied mast cell density in pediatric age group on rather larger number of cases in a referral children hospital with longstanding experience in pediatric gastroenterology [13]. Mast cell density was 12.6 ± 0.87 in 0.25 mm2 (range: 0-81) in our study.

We found no significant correlation between mast cell density and sex of patients. Also no significant correlation was found between mast cell density and presence and degree of inflammation, activity and presence of H. pylori [13]. However we proposed that maybe mast cells play a role in producing clinical symptoms not by their traditional known pathways in inflammatory processes but by an as yet undefined pathway such as stimulatory effect on gastric nerve plexuses. So we recommended that increase in mast cell density should be included in the final report of gastric biopsies [13].

We frequently encounter cases with rather normal gastric biopsies or only mild chronic inflammation with no H.pylori, which mainly complain of chronic abdominal pain. We gathered these cases with mast cell density more than 30/0.25 mm^2 ^from 895 gastric biopsies and wanted to study their clinical and endoscopic findings and propose a new entity.

## Materials and methods

Between April 2005 and May 2008, 895 children (< 14 years old), with gastrointestinal complaints who underwent endoscopy were selected and antral biopsies were obtained for histological examination at Children Medical Center (children hospital affiliated with Tehran University of Medical Sciences). The specimens were fixed in 10% buffered formalin, processed, embedded in paraffin and cut in sequential 3 micrometer sections. Superficial and deep sections were stained by Hematoxylin-eosin (two slides) and one slide was stained by Giemsa stain. Mast cells were counted by Giemsa stain at ×1000 magnification in 10 fields with a Zeiss standard 20 light microscope and the sum was calculated for each case (measuring 0.25 mm^2^). All the mast cell counts and histological evaluation was performed by a single observer (F. Mahjoub).

Among these children, those who had normal or erythematous (but not nodular or ulcerative) gastric mucosa on endoscopic view, plus pathologic report of normal mucosa or mild gastritis in addition to mast cell count more than 30/25 mm^2^, were chosen and a questionnaire was filled for each patient including clinical, endoscopic and pathologic findings.

The statistical analysis was performed using SPSS, version 13 (SPSS Inc., Chicago, IL, USA).

## Results

Over a 3 year period of study, of 895 selected children, 86 patients fulfilled the entrance criteria. Among these patients, 55.8% (48 persons) were male. There was no significant difference between sex of patients (p > 0.05). The mean age of patients included in the study was 6.68 ± 3.06 (range: 1-14 years). Table 1 provides a summary of patients' clinical manifestations. As shown in table 1, most common clinical manifestations were recurrent abdominal pain (n = 59 (68.6%)) and vomiting (n = 34 (39.5%)). No significant relation was found between sex and type of gastrointestinal complaints (p > 0.05). Endoscopic and pathologic findings are shown in table 2. Non of our patients had signs of systemic mastocytosis.

**Table 1:** Frequency of clinical manifestations

**Clinical manifestation **	**Frequency**	**%**
Recurrent abdominal pain	59	68.6
Vomiting	34	39.5
Low weight gain	17	19.8
Gastrointestinal bleeding		
Hematemesis	7	8.1
Bloody stool	3	3.5
Gastro esophageal reflux disorder	6	7
Heart burn	2	2.3
Diarrhea	2	2.3
Anemia	2	2.3
Chronic renal failure	2	2.3
Others	4	4.7

Total	86	100

**Table 2:** The relationship between endoscopic and pathologic findings

		**Gastric pathology**			**Total**
		**Normal**	**Mild gastritis**	**Decrease in glands' number and fibrosis**	

Gastric endoscopy	Normal gastric mucosa	25 (29.1%)	11 (12.8%)	3 (3.5%)	39 (45.3%)
	Diffuse erythema	10 (11.6%)	7 (8.1%)	1 (1.2%)	18 (21%)
	Patchy erythema	15 (17.4%)	11 (12.8%)	0 (0%)	26 (30.2%)
	Localized fundal erythema	1 (1.2%)	0 (0%)	0 (0%)	1 (1.2%)
	Missing	1 (1.2%)	0 (0%)	0 (0%)	1 (1.2%)
	Edematous gastric mucosa	1 (1.2%)	0 (0%)	0 (0%)	1 (1.2%)

Total		53 (61.6%)	29 (33.7%)	4 (4.7%)	86 (100%)

**Figure 2:**
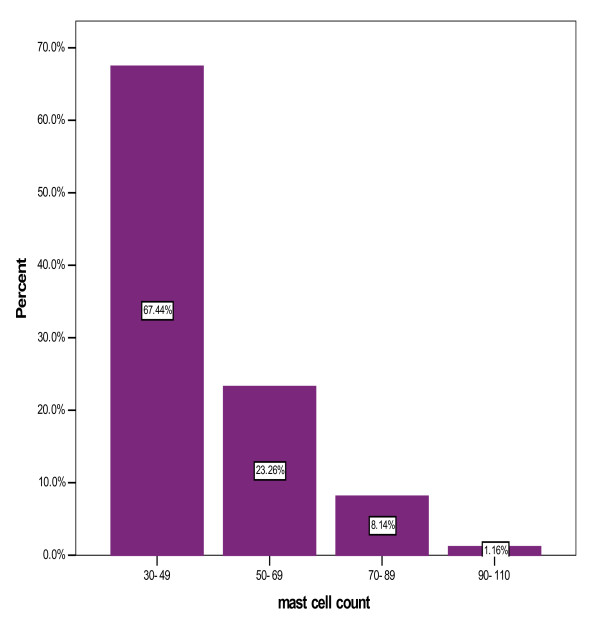
**Distribution of mast cell density**.

**Figure 3:**
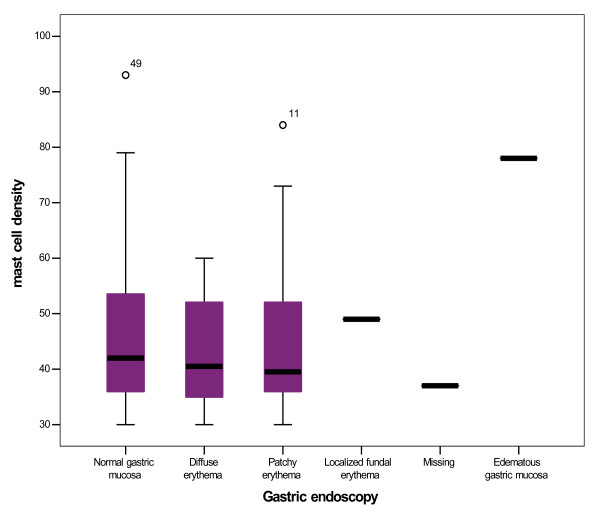
**Box plot of mast cell density and gastric endoscopy**.

Mast cell density was divided in four categories: [30-49], [50-69], [70-89] and [90-110]. The mean mast cell density was 45.59 ± 13.81 in 0.25 mm^2 ^(range: 30-93). Among our cases, about 67.4% (n = 58) had 30 to 49, 23.3% (n = 20) had 50 to 69, 8.1% (n = 7) had 70 to 89 and 1.2% (n = 1) had 93 mast cells/0.25 mm^2 ^in their specimens (figure 2). No significant relation was found between clinical and endoscopic findings and mast cell density (p > 0.05) (figure 3).

As shown in table 3, of 86 cases, 61.6% of gastric biopsies showed no significant pathologic change, 33.7% mild chronic gastritis and 4.7% decrease in glands' number and fibrosis.

**Table 3:** Mast cell density and pathological findings

		**Gastric pathology**			**Total**
		**Normal**	**Mild gastritis**	**Decrease in glands' number and fibrosis**	

Mast	30-49	36	18	4	58
Cell	50-69	13	7	0	20
Density	70-89	4	3	0	7
	90-110	0	1	0	1

Total		53	29	4	86

## Discussion

Results of some studies suggested that mast cells play a role in the inflammatory reaction in the course of gastritis. There is only little data in literature concerning the number and role of mast cells in children with gasterointestinal complaints and existing data have rather conflicting results.

Mast cell detection is rather easy by a simple and inexpensive staining (Giemsa) which can also be used to detect presence of H. pylori. It is of worth nothing that reliable detection is achieved by examining the tissue under oil immersion (×1000) and in lower magnifications they can be missed. Although the gold standard is staining of tissue with anti-tryptase antibody by immunohistochemical methods, it is a time consuming and rather expensive method and is not recommended for routine pathologic assessment of gastric biopsies [13].

In this study, we evaluated biopsy specimens of gastric mucosa collected from pediatric patients in a referral children hospital with longstanding experience in pediatric gastroenterology who had normal histology except increase in mast cells.

The mean mast cell density of our cases was 45.59 ± 13.81 in 0.25 mm^2^(range: 30-93).

In 29% of our cases, neither endoscopic nor pathologic change was detected and only increase in mast cell number was reported and in others endoscopic and histopathological findings were negligible except increase in mast cells. In updated Sydney system (classification and grading of gastritis) [14], no term is introduced which is in concordance with this group. We propose that maybe mast cells play a role in producing such symptoms not by their traditional known pathways in inflammatory processes but by an as yet undefined pathway such as stimulatory effect on gastric nerve plexuses [13]. Mast cell count is not routinely performed in gastric biopsies, however rough estimate of mast cell density can be done in Giemsa stained slides. We recommend that mast cell density should be included in the final report of gastric biopsies. We also propose the term "mast cell gastritis" as a new and different term to be included in classification and grading of gastritis, in cases with normal histology but mast cell count above 30/25 mm^2^.

We think that increased density of mast cells in these cases should not be overlooked and it may contribute to clinical manifestations in some way. We hope that further studies will direct us to institute therapeutic measurements in this regard.

## Competing interests

The authors declare that they have no competing interests.

## Authors' contributions

FM carried out the pathologic studies and drafted the manuscript. FF carried out the endoscopic studies and helped in pediatric gastrointestinal aspects. ZP participated in the immunological aspects of our article. HA and ZA helped in collecting the data, methodological aspects and design of article. All authors read and approved the final manuscript.
